# Wilms’ Tumor in Adults—Conventional and Unconventional Presentations of a Rare Entity with a Review of Literature

**DOI:** 10.15586/jkcvhl.v8i2.186

**Published:** 2021-07-20

**Authors:** Sujata Tripathi, Amit Mishra, Vijay C. Popat, Syed Altaf Husain

**Affiliations:** 1 Department of Pathology & Blood cell, Rana Beni Madhav District Hospital, Raebareli, India;; 2 Department of Urology, AIIMS, Raebareli, India;; 3 Department of Pathology, MP Shah Government Medical College, Jamnagar, India;; 4 Department of Radio Diagnosis, Rana Beni Madhav District hospital, Raebareli, India

**Keywords:** adult Wilms’ tumor, inferior vena cava, nephroblastoma, NWTS, SIOP, thrombus

## Abstract

Wilms’ tumor (WT) in adults is a rare neoplasm. Only a few reports are available in the literature. The tumor often masquerades as renal cell carcinoma (RCC). For accurate reporting, histopathological examination (HPE) plays a vital role in early diagnosis and prompt administration of multimodality treatment helps to improve the prognosis.

We comprehensively analyzed five cases of adult WT presenting in the third to fifth decade with flank pain, hematuria, fever, and palpable lump. After complete clinical, biochemical, radiological, and HPE evaluation, tumor was staged and treatment was planned accordingly.

Patients with low-stage WT were treated with open radical nephrectomy and chemotherapy. One of the patients diagnosed with inferior vena cava (IVC) thrombus apart from the above treatment also underwent IVC thrombectomy. Another young male presenting with distant metastasis (stage IV) and focal anaplasia on histology received preoperative chemotherapy and then planned for surgery. Unfortunately, the tumor being unresectable, second-line chemotherapy was given but he ultimately succumbed to death. All other patients are on regular follow-up and disease-free.

Adult nephroblastoma is a rare clinical entity with hostile behavior. The presence of IVC thrombus is not a contraindication to surgery. Although the management strategy as per pediatric protocol by the inclusion of multimodality approach improves survival, still the overall prognosis in adults is dismal. There is a need for a standardized treatment protocol to encourage a homogenous approach for this rare disease and thereby improve survival.

## Introduction

Wilms’ tumor (WT), presumably has originated from primitive metanephric blastema and is named after Carl Max Wilhelm Wilms, noted German surgeon of the 19th century (1). It is the most common malignant renal tumor in childhood but is extremely rare in adults with an estimated incidence of only 0.2 cases per million. Only 1% to 2% of patients have a family history (2). Large series of adult patients are rare (3, 4), presenting stage in adults is often higher and clinical course more hostile as compared to children. They relapse more frequently and respond poorly to therapy. This study is an effort to highlight the myriad clinical presentations, diagnostic as well as technical challenges; and most importantly, the paucity of standard management guidelines in the treatment of such patients requiring a multimodal treatment approach.

## Subjects and Methods

This clinical study was conducted in a tertiary care hospital over a decade from June 2010 to August 2020. After approval from the Ethics Committee and taking informed consent, patients were enrolled for the study.

An extensive review of the literature was done from articles published over the last 25 years. Various search engines were used to identify relevant articles. The keywords: nephroblastoma, adult wilms tumor, outcome, inferior vena cava (IVC), thrombus, National Wilms Tumor Study (NWTS) group, International Society of Pediatric Oncology (SIOP) were used. With this background, we analyzed five adult Wilms’ tumor patients presenting in the third to fifth decade of life. Four patients were male and one of them was a female. The most common presentation being flank pain, hematuria, fever, and palpable lump (Table 1). All patients were evaluated with complete history, physical examination, and blood biochemistry (complete blood count, urine examination, renal and liver function tests, serum electrolytes). Radiological evaluation with contrast-enhanced computed tomography (CECT) and magnetic resonance imaging (MRI) of abdomen and pelvis as and when indicated. Additional studies were done in a patient with IVC thrombus to evaluate cardiac and respiratory status.

**Table 1:** Showing the clinical, radiological, therapeutic, and histopathological details of the cases included in our study.

Case no.	1	2	3	4	5
**Clinical features**	34/M, pain, fever, hematuria, and palpable lump–3 months	25/M, painless lump, abdominal discomfort, vomiting – 4 months	37/F, Pain, hematuria, hard palpable lump – 6 months	40/M, Pain, hematuria, B/L pedal edema, anemia – 3 months	24/M, Pain, hematuria, weight loss, decreased KPS, palpable lump—5 months
**Radiological findings**	RK, LP mass (19 × 13 × 22 cm), heterogenous hypointense on T2WMRI	RK, LP mass (17 × 16 × 12), calcification, hypodense on CT No contrast enhancement	LK, UP mass (13 × 12 × 10cm), hypodense, heterogenous, with thin peripheral enhance ment on CT	LK, MP & LP mass (10 × 6 × 7), hyperint ense, heterogenous on T2W MRI, with renal vein and IVC thrombus (Level II)	L.K., (16 × 15 × 16 cm) lobulated, heterogenous, hypodense mass on CT, multiple lymph nodes, few liver and lung mets
**Treatment**	Right open radical nephrectomy followed by chemotherapy	Right open radical nephrectomy followed by chemotherapy	Left open radical nephrectomy followed by chemotherapy	Left open radical nephrectomy with IVC thrombectomy and regional lymphadenectomy followed by chemotherapy	Preoperative chemotherapy but found unresectable, so second-line chemotherapy, succumbed to death.
**Histopathology**	Triphasic, WT1 +ve, stroma vimentin +ve, CD 10-ve, No anaplasia.	Marked calcifica tions, triphasic, vimentin +ve, WT1 +ve. No anaplasia.	Triphasic Wilms, WT1 +ve cells, vimentin +ve. No anaplasia	Highly cellular with predominant blastemal elements, spindle cells WT1 +ve, Vimentin +ve, No anaplasia	Triphasic pattern, predominant undifferentiated blastemal cells WT1 +ve, vimentin +ve with focal anaplastic cells
**ECOG**	0	0	0	1	2
**Stage**	I	I	I	II	IV
**Clavien Dindo complications**	I	Nil	II	II, IIIa	–
**Follow up (months)**	Lost to FUP	84	On chemo since 4 months	60	Mortality

RK: Right kidney; LK: Left kidney; M: Male; F: Female; KPS: Karnofsky performance score; B/L: Bilateral; UP: Upper pole; MP: Midpole; LP: Lower pole; MRI: magnetic resonance imaging, CT: Computed tomography; IVC: Inferior vena cava; ECOG: Eastern Cooperative Oncology Group; chemo: chemotherapy; FUP: Follow up.

## Surgical Technique

All patients were operated under general anesthesia, with modified chevron incision commencing approximately two fingerbreadths below the costal margin and extending laterally to the midaxillary line. The kidney was mobilized laterally and posteriorly, and the perirenal collateral circulation was ligated. Then renal artery was identified, ligated, and divided, consequently the collateral circulation collapsed making the rest of the dissection easier (5, 6).

In case 4 with IVC thrombus, a plane was then created between the IVC and posterior abdominal wall, small tributaries were identified and ligated. The advantage of creating this plane was to facilitate circumferential control of the IVC. The use of self-retaining liver retractor was of immense help and made the surgery quite effortless. Vascular isolation of the IVC was achieved by placing vascular clamps in the following order: caudal IVC first, then the right renal artery, right renal vein, and cephalic IVC. Extraction of the thrombus was facilitated by a 2–3 cm longitudinal incision on the IVC beginning at the level of the renal vein and extending cranially, encompassing a vessel wall rim of the orifice of the resected renal vein. The tumor thrombus was then “milked” downwards out along with the whole kidney specimen. After extraction of the specimen, the IVC was repaired in a bloodless surgical field with a continuous polypropylene 5-0 suture.

Detailed histopathological examination (HPE) was performed in all cases and immunohistochemistry was done as indicated. The staging of adult WT was done similar to pediatric tumors in accordance with NWTSG.

All patients were subjected to multimodality treatment according to the stage and histology. Strict follow-up was done with clinical assessment, blood biochemistry, and CT scan of chest, abdomen ,and pelvis biannually for 3 years followed by annual scans thereafter.

## Result

Out of five patients, three patients with low-stage disease (Case 1, 2, 3) and favorable histology underwent open radical nephrectomy, mean duration of surgery was around 110 min and the mean blood loss being 450 ml. Case 1 had a fever and wound infection, Case 3 had paralytic ileus which resolved with conservative management, Case 4 needed a blood transfusion in the postoperative period and had wound infection and dehiscence, apart from that postoperative period was uneventful. Each patient received standard adjuvant chemotherapy. Cases 1, 2, and 3 with stage I favorable histology adult WT received two drugs vincristine (1.5 mg/m^2^) and actinomycin D (15 µg/kg) regimen for 18 weeks. Case 4 with stage II favorable histology received three drugs regimen comprising of vincristine (1.5 mg/m^2^), actinomycin D (15 µg/kg), and doxorubicin (50 mg/m^2^) for 24 weeks.

Case 5, a young male with metastatic disease, underwent an initial biopsy which demonstrated unfavorable histology, so upfront chemotherapy was given for 6 weeks, with vincristine 2 mg per week and doxorubicin 50 mg/m^2^ (d1 = d28). The patient was then planned for radical nephrectomy followed by adjuvant chemotherapy and radiation, but intraoperatively tumor was unresectable. Second line of chemotherapy that consisted of etoposide 200 mg/m^2^ for 3 days and cisplatin 100 mg/m^2^ (d1 = d21) was started. After three cycles, the CT scan showed no response to therapy. Within 15 days, patient’s performance status deteriorated and he died 3 weeks after the last cycle.

Case 4 in our study, with renal mass and IVC thrombus, underwent left open radical nephrectomy with IVC thrombectomy and regional lymphadenectomy, duration of surgery was around 200 min and blood loss was 900 mL. In the postoperative period, he required blood transfusion and secondary suturing of the wound. He was discharged on day 10 with a plan for chemotherapy.

## Discussion

The most common presenting symptom in adults includes pain (abdominal, flank, or back) in 50%–80% of cases, followed by signs like hematuria and abdominal mass in about 30%–60% of patients. Fever, weakness, and weight loss being less common. Seldom adult WT is an incidental finding (7) or diagnosed because of paraneoplastic or tumor-induced erythrocytosis (8).

Due to identical imaging characteristics, it is difficult to differentiate a WT from renal cell carcinoma (RCC) in adult patients. Wu et al. (9), in their study, described WT as attenuation of less or equal compared to renal parenchyma on unenhanced CT (P > 0.05), while tumor enhancement after contrast administration was lower than that of normal renal parenchyma (P < 0.05). In all three cases where CECT was done, the tumor was hypodense.

**Figure 1:**
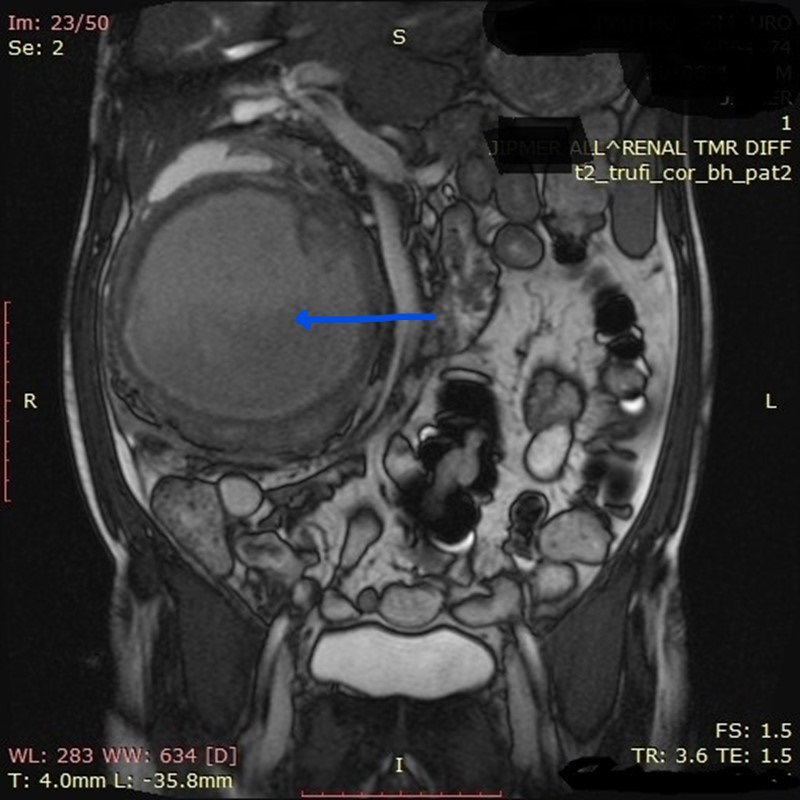
MRI of the abdomen and pelvis showing heterogenous hypointense large right renal mass, arising from lower pole, fat plane maintained with liver, IVC compressed by mass and pushed laterally.

**Figure 2:**
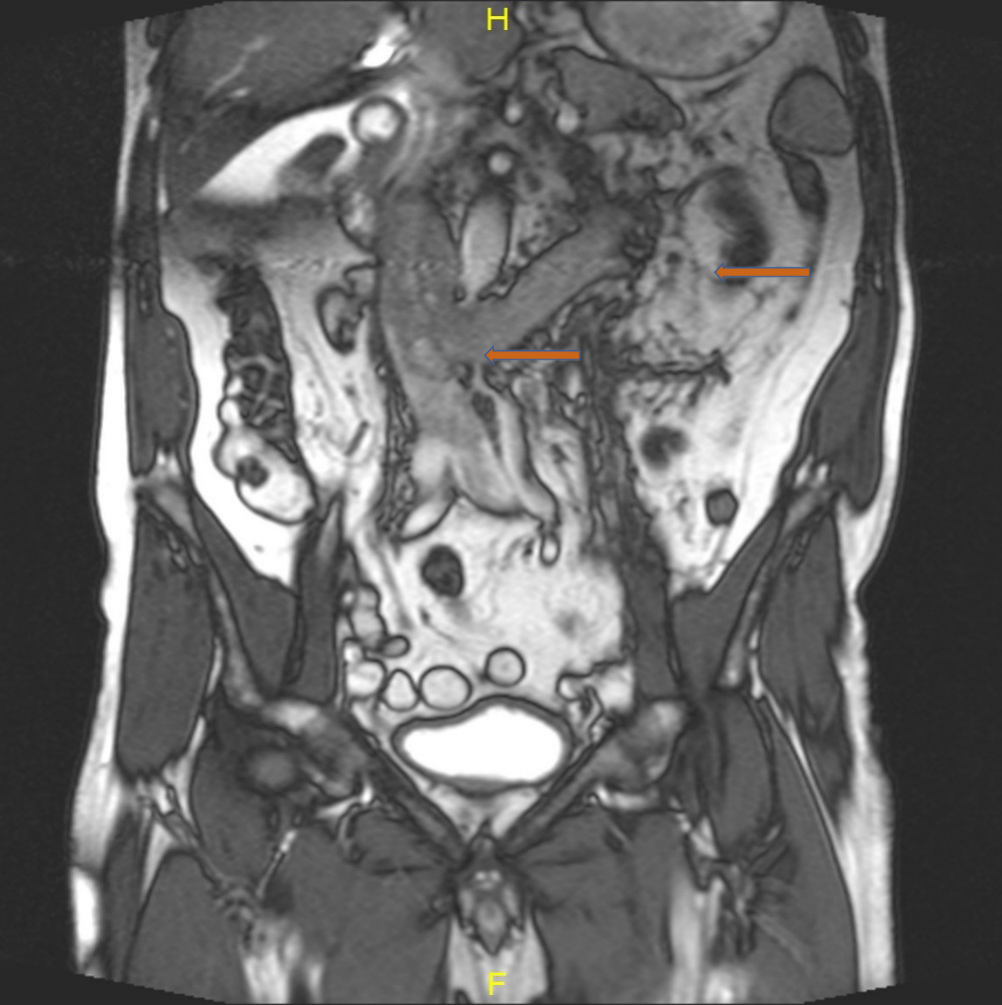
MRI of the abdomen and pelvis showing left renal mass, renal vein, and IVC thrombus.

On T2W MRI it is described as an isointense or hypointense signal in all phases in distinction to many renal tumors (which are usually hypervascular) that are heterogeneous and hyperintense on T2W imaging. Therefore, one may distinguish between WT and tumors with hypervascularity based on differences in enhancement (9). Out of two cases (1 and 4) where MRI was done, in the first patient, tumor was typically heterogenous and hypointense (Figure 1) while in the fourth patient with IVC thrombus tumor, it was unusually hyperintense (Figure 2). MRI is the gold standard for (i) staging renal tumors; (ii) delineating the level and extent of tumor thrombus in the IVC, besides being exceptionally useful in excluding caval wall invasion altogether playing a vital role in precise planning of surgery. It has a greater edge over CT for thrombus detection, delineating its upper level and staging of renal tumor owing to its ability to have a free imaging plane with an optimal spatial resolution in the sagittal and coronal planes (10).

**Figure 3:**
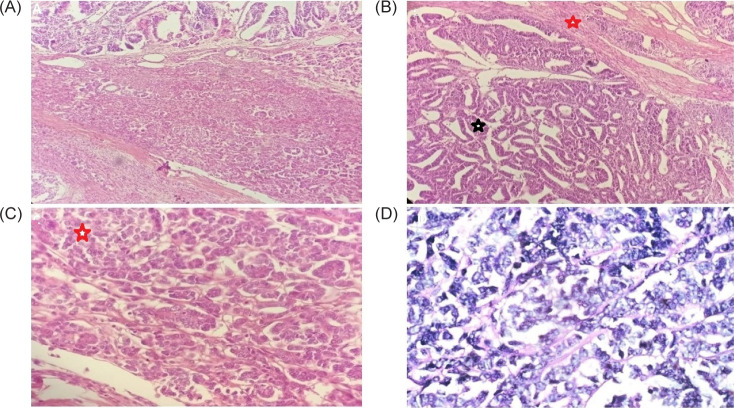
(A) Histopathological examination (HPE) of specimen from first patient showing features of Wilms’ tumor, 40× magnification. (B) HPE of specimen from second patient showing epithelial and stromal component of Wilms’ tumor. Black star highlights epithelial component and red star highlights stromal component, 100× magnification. (C) HPE of specimen from third patient showing blastemal component highlighted by red star, 200× magnification. (D) HPE of specimen from fourth patient showing only blastemal component, 400× magnification.

Wilms’ tumor in adults and children have similar histopathological characteristics. The WT1 gene (11p13) is mutated in 10% of tumors. 11p, 7p, 16q, and 1p alterations are also documented. In our study, no genetic markers were tested for classical triphasic histopathological pattern comprising of blastemal, epithelial, and stromal elements is the hallmark (Figure 3A, B, and C). Blastemal-predominant WTs are more hostile and have a poorer prognosis as seen in our fourth case where the tumor had invaded IVC (Figure 3D). A blastema is regarded as the least differentiated, and most malignant, component and consists of small, round blue cells with overlapping nuclei and brisk mitotic activity. Pure blastemal-type WTs have to be differentiated from other poorly differentiated tumors, such as neuroblastoma, primitive neuroectodermal tumor, and Ewing sarcoma of the kidney. Since blastemal predominant features have been considered a high-risk pathological finding, intensive chemotherapy was administered to these patients according to SIOP protocol. However, according to NWTS protocol, there has not been a single case report depicting that a predominancy of blastemal cells contributes to a poorer prognosis. Beckwith et al. (11) reported that the diffuse blastemal pattern was associated with marked aggressiveness, but with a high survival rate due to the good response to chemotherapy. Epithelial and stromal variants are characterized as intermediate-risk tumors (12). In our study, three patients with initial stage disease, above mentioned classical HPE findings were present along with no anaplasia.

Patients with anaplasia have a very aggressive course of the disease as seen in case 5, early age of presentation with clinical and radiological features of advanced disease (stage IV). His Trucut biopsy revealed triphasic pattern with predominant blastemal cells and focal anaplastic cells. He received preoperative chemotherapy, in spite of it, his tumor was not amenable for surgery and later succumbed to death.

Anaplasia as identified by multipolar mitotic figures, enlarged nuclei at least three times and hyperchromatic nuclei, is reported in 10% of WT cases. Its recognition and documentation as “focal or diffuse” are very essential. Diffuse anaplasia cases have a higher relapse rate and poorer prognosis as compared to focal anaplasia (13, 14). This case of stage IV WT clinches the association of aggressive clinical behaviour with the above histopathological findings.

Adult WT is diagnosed based on the criteria given by Kilton et al. (15). These include (i) the tumor under consideration should be a primary renal neoplasm; (ii) presence of primitive blastemic spindle or round cell component; (iii) formation of abortive or embryonal tubules or glomerular structures; (iv) no area of tumor diagnostic of RCC; (v) pictorial confirmation of histology and (vi) patient’s age >15 years.

Masuda et al. (16) have suggested that calcification may be a sign of slow tumor growth and possibly indicate a favorable prognosis in cases of adult WT. Calcified tumors may be relatively large, tend to be localized, and histologically well-differentiated. Case 2 in our study had marked calcifications and the patient is doing well to date with no relapse.

**Figure 4:**
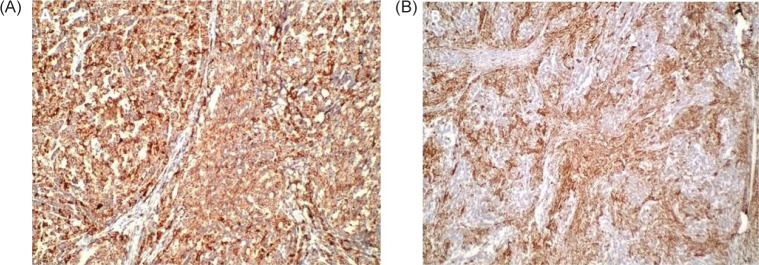
(A) Tumor cells are positive for vimentin on immunohistochemistry, 10× magnification. (B) Tumor cells are positive for WT-1 on immunohistochemistry, 10× magnification.

Additional diagnostics such as immunohistochemical staining for the presence of cytokeratin, vimentin (Figure 4A), desmin, actin, and WT1 (Figure 4B) allows distinguishing between other rare cancer types such as renal sarcoma, mesoblastic nephroma, clear cell sarcoma, or rhabdoid tumor. The WT1 expression is diagnosed in the blastemic area and proliferating epithelial tissue, but not in the mature stroma and mature epithelial tissue (4).

Another interesting and rare case (case 4) of a 40-year-old male with clinical and radiological features suggestive of renal mass with venous occlusion secondary to tumor thrombus extending into renal vein and IVC (Level II), a clinical diagnosis of left RCC with IVC thrombus was made. Histopathological evaluation inadvertently revealed findings (triphasic pattern with predominantly blastemal component) consistent with aggressive adult WT.

The IVC tumor thrombus may be present in 4%–10% of patients of adult WT identical to RCC but the exact incidence of venous involvement in adult WT is seldom reported and still unknown (17). These cases are extremely challenging for a surgeon. Martínez-Ibáñez et al. (18) suggested that surgical strategy for these cases depends on the length of the thrombus. If the thrombus can be easily removed, complete resection is the treatment of choice and improves the prognosis (3), even in the presence of distant metastases. Our patient received postoperative chemotherapy and had a progression-free survival thereafter.

In patients with thrombus, (i) invading the wall of the IVC, (ii) extending to intrahepatic IVC, supra hepatic IVC level, or the right atrium, preoperative chemotherapy is beneficial and recommended. In our case, thrombus was limited to infrahepatic level, so preoperative chemotherapy was not offered.

As it is a rare disease in adults, standard treatment protocol has not been defined to date. There is a common consensus that a multimodality approach is required in the treatment, involving surgery, chemotherapy, and/or radiotherapy.

Two approved protocols for management are SIOP and NWTS. SIOP suggests chemotherapy prior to nephrectomy for tumor shrinkage and intraoperatively less chance of spillage. It also downgrades the tumor, so the overall treatment required is less whereas NWTS recommends upfront nephrectomy to decide further treatment based upon histology and stage of tumor (Table 2) (19, 20).

**Table 2:** Management as per NWTS protocol.

Management as per NWTS protocol		
Stage	Histology	Treatment
I	Favorable	18 weeks of ACT/VCR
	Unfavorable	
II	Favorable	18 weeks of ACT/VCR
III	Favorable	24 weeks of ACT/VCR/ADM, RT tumor bed + involved sites
IV	Favorable	
II–IV	Unfavorable	24 weeks of ACT/VCR/ADM/CTX/Etoposide, RT tumor bed + involved sites

ACT: Actinomycin D; VCR: Vincristine; ADM: Adriamycin (Doxorubicin); RT: Radiotherapy; CTX: Cyclophosphamide; NWTS: National Wilms Tumor Study group.

### Surgery

Radical nephrectomy is the gold standard treatment. Nephron sparing surgery is recommended only in some cases (bilateral tumor, congenital anomaly in the other kidney, solitary kidney) as per SIOP (21).

### Chemotherapy

The SIOP recommends preoperative chemotherapy to decrease the incidence of local and distant recurrence. Some potent drugs used are actinomycin D (ACT), vincristine (VCR), doxorubicin (ADM), cyclophosphamide (ctx), ifosfamide (IFO), etoposide, and carboplatin (as in monotherapy drug combination) (22, 23). NWTS endorses adjuvant chemotherapy with ACT, VCR, ADM for 24 weeks in stage III disease while less aggressive treatment with two drugs (VCR and ACT) in stages I and II. Risk characteristics that are indications for adjuvant chemotherapy include (i) age at the presentation—adult age group presentation in all stages; (ii) unfavourable histology-focal or diffuse anaplasia; (iii) patients with LOH 1p/16q (1, 3).

### Radiotherapy

Wilms’ tumor is radiosensitive and according to various SIOP trials, it prevents tumor rupture as well as downgrades the tumor stage. Existing guidelines recommend radiotherapy only for advanced WT (stages III, IV, and V) and low-stage tumors with unfavorable histology. NWTS and SIOP recommended doses are 10, 15, and 20 Gy, respectively (20).

The NWTS group documented an overall survival rate of 82% in favorable histology of adult WT (24). Reinhard et al. (25) reported an overall survival of 83% and inferred that most of the adult WT cases can be treated by a multimodal approach identical to pediatric protocols. Terenziani et al. (26) also arrived at a similar conclusion in 17 cases. Several case series have proposed tremendous improvement in prognosis when pediatric treatment protocols including multimodal strategy are utilized (27, 28).

We reviewed the literature and analyzed the clinical, pathological, treatment, and outcome parameters in various studies and compared these variables with our findings (Table 3).

**Table 3:** Review of literature illustrating comparable clinicopathological treatment and outcome variables.

Study groups →	Reinhard et al. (2004)	Mitry et al. (2004)	Izawa et al. (2008)	Kalapurkal et al. (2004)	Kattan et al. (1994)	Our study (2010–20)
Variables ↓						
**Mean age (years)**	25.4	34	26	21.9	24	32
**Total No.** **(M/F)**	30 (17/13)	133 (64/69)	128	23 (10/13)	22 (8/14)	5 (4/1)
**Clinical features**	Pain, palpable lump, gross hematuria	NIA	NIA	NIA	Pain, hematuria, abdominal mass	Pain, hematuria, abdominal mass
**Laterality**	18 Rt:10 Lt, 2 Extrarenal	NIA	NIA	NIA	Rt (14), Lt (8)	Rt(2), Lt(3)
**Stage**	66% localized, 33% metastatic	23% -loco regional, 10% metastatic, 66%-NIA	NIA	Stage I/II-13, Stage≥III-10	Stage I/II-12, Stage≥III-10	Stage I-3 Stage II-1 Stage IV-1
**Treatment**	Surgery only (3), Neoadjuvant CT (4)	NIA	Surgery, CT (120), CT+RT (77), No T/t- 7, NIA -1	Surgery, CT only (10), CT + RT (13)	Multimodal (15), Adjuvant CT (6), Adjuvant RT (1)	Surgery (4), CT (5)
**HPE**	Blastemal predominant (M.C.), Mixed (CCSK), Stromal or epithelial	NIA	NIA	NIA	FH (21), UFH (1)	FH (4), UFH (1)
**Median FUP (months) &** **[O.S.]**	48 [83%]	60 [47%]	54 [68%]	61 [82%]	100 [55%]	120 [1-lost to FUP, 1 expired, 1 on CT, rest in complete remission]
**Mortality**	5	NIA	NIA	NIA	10	1

Rt: Right; Lt: Left; NIA: No information available; CT: Chemotherapy; RT: Radiotherapy; T/t: Treatment; M.C.: Most common; CCSK: Clear cell sarcoma of kidney; M/F: Male/Female; FUP: Follow up; FH: Favorable histology; UFH: Unfavorable histology; HPE: Histopathological examination; O.S.: Overall survival.

## Follow Up

Long-term follow-up of these patients is of utmost importance because of the risk of relapse and potential side effects of chemoradiation. In our case series, the patient with IVC thrombus received postoperative chemotherapy and is having a disease-free survival at 5th year of follow-up. Amongst the remaining three patients with low-stage disease, one was lost to follow-up, one is still on chemotherapy, and another is on regular follow-up and disease-free to date.

## Conclusions

Adult WT is a rare entity with mainly case reports in literature. The likelihood of WT should be kept in mind in any adult presenting with flank pain, large tumor mass, and aggressive growth. Being so rare, the diagnosis is made solely on histopathology with hallmark triphasic pattern. From our study, we concluded that there are no established protocols for treatment, still it is curable if treated according to pediatric strategy, including multimodal approach. The presence of IVC thrombus along with its parasitic vasculature and multiple collaterals make the procedure technically challenging but is not a contraindication for surgery and can be managed safely with improved surgical techniques.

All WT patients should be registered to international databases which will immensely assist in the research and development of future management guidelines for this rare entity.
